# Brownian Motion Simulation for Estimating Chloride Diffusivity of Cement Paste

**DOI:** 10.3390/ma16052002

**Published:** 2023-02-28

**Authors:** Congyan Zhang, Xiang Li, Feng Chen, Xudong Wang, Jianjun Zheng

**Affiliations:** 1 Yuanpei College, Shaoxing University, Shaoxing 312000, China; 2 School of Architecture and Engineering, Zhejiang Industry Polytechnic College, Shaoxing 312000, China; 3 School of Civil Engineering, Zhejiang University of Technology, Hangzhou 310023, China

**Keywords:** chloride diffusion coefficient, cement paste, Brownian motion, numerical simulation

## Abstract

Chloride ion diffusion properties are important factors that affect the durability of cementitious materials. Researchers have conducted much exploration in this field, both experimentally and theoretically. Numerical simulation techniques have been greatly improved as theoretical methods and testing techniques have been updated. Researchers have modeled cement particles mostly as circular shapes, simulated the diffusion of chloride ions, and derived chloride ion diffusion coefficients in two-dimensional models. In this paper, a three-dimensional random walk method based on Brownian motion is employed to evaluate the chloride ion diffusivity of cement paste with the use of numerical simulation techniques. Unlike previous simplified two-dimensional or three-dimensional models with restricted walks, this is a true three-dimensional simulation technique that can visually represent the cement hydration process and the diffusion behavior of chloride ions in cement paste. During the simulation, the cement particles were reduced to spheres, which were randomly distributed in a simulation cell with periodic boundary conditions. Brownian particles were then dropped into the cell and permanently captured if their initial position in the gel fell. Otherwise, a sphere tangential to the nearest cement particle was constructed, with the initial position as the center. Then, the Brownian particles randomly jumped to the surface of this sphere. The process was repeated to derive the average arrival time. In addition, the diffusion coefficient of chloride ions was deduced. The effectiveness of the method was also tentatively confirmed by the experimental data.

## 1. Introduction

The presence of chloride in service environments is the main factor that causes the corrosion of steel bars in cementitious materials, which seriously affects the endurance of concrete structures [1,2,3,4,5]. Researchers around the world have made extensive efforts to investigate the mechanism of chloride ion invasions in cementitious materials.

Regarding experimental studies, the following are worthy of note: MacDonald et al. [6] investigated the influence of the water-to-cement ratio (*w*/*c*) as well as additional test conditions, such as sample thickness and chloride ion concentration, on the identification of the cement paste diffusivity of chlorine. Their results show that the chloride ion concentration had no distinct effect on the diffusivity of chlorine; however, the measurement time of the diffusion coefficient decreased as the concentration increased. Furthermore, the results show that an increase in thickness slightly decreased the effective chloride ion diffusion coefficient. This method had strict requirements for temperature and humidity control, which were difficult to meet, along with an excessively long test cycle. Ngala and Page [7] determined the steady-state diffusion energy of chloride ions, the size distribution of the pores, total porosity, and coarse capillary porosity. It was found that pre-drying and carbonating the cementite adversely affected the diffusion resistance of ions, especially for blended cements. However, in addition to its long test period, this method had many influencing factors that were difficult to control. Lu [8] proposed the use of the Nernst–Einstein equation (NEL method) to determine the chloride diffusion coefficients of cement-based materials. Taking cementite as a solid electrolyte, the relationship between the diffusion coefficient and conductivity was obtained by applying the Nernst–Einstein equation. Ampadu et al. [9] used the accelerated chlorine diffusion test to enhance the traditional test method. They found that the chlorine diffusion rate of the early-age cement paste was heavily influenced by the *w*/*c*, and that the diffusion rate was considerably reduced with the addition of fly ash. A comparison of natural diffusion and electric-field-accelerated migration tests showed that they had similar magnitudes, which confirmed that the steady-state migration test can be used instead of the steady-state natural diffusion test at a specific voltage [10]. The average depth of chloride penetration and chloride migration coefficient in concrete were estimated by the rapid chloride migration test (RCMT) and accelerated chloride migration test (ACMT) [11]. From the experiments, it was observed that the chloride diffusivity increased with the increase in water–cement ratio, and RCMT obtained more penetration depth compared with ACMT. To study the impact of porosity on the chlorine diffusion coefficient, Pivonka et al. [12] performed a series of experimental data analyses. They derived the correlation between diffusivity and microscopic porosity as well as pore solution chloride diffusivity by comparing the chloride diffusivity of cementite samples at different porosities, which were created with different *w*/*c*. However, the effect of isolated pores on cementite was not considered, which caused an overestimation of the chloride diffusivity. Yang et al. [13] obtained the relationship between pore structure and chloride diffusivity from a ponding test in cement-based materials. Maes et al. [14] investigated the effect of partial replacement of ordinary Portland cement (OPC) with blast furnace slag (BFS) on the chloride resistance of concrete by comparing the chloride profiles and color change boundaries after non-steady state migration and natural diffusion tests. Their results indicated that the addition of BFS reduced the chloride binding capacity of concrete. For high-volume fly ash (HVFA) concrete, higher temperature at early age was beneficial for the development of performances [15]. High temperatures also favored the resistance of alumina-rich slag cements to chloride penetration [16]. Chalabi et al. [17] found that chlorine translocation was dependent on saturation, provided that the chloride content of the material increased while its moisture content decreased from 97% to 76%. Ramirez-Ortiz et al. [5] detected the appearance and combination of chloride ions within cement pastes using ultrasound. The results show that the energy/amplitude weighted-average frequency had an index that was dependent on the amount of chemically bound chloride in the cement pastes. Millar et al. [18] identified the total chloride content of cement paste with the use of laser-induced breakdown spectroscopy. Zhang et al. [19] detected the presence of chlorine in cement with a developed collinear dual-pulse LIBS system, which increased the assay sensitivity of the trace-elemental chloride in the cement paste. Their findings indicate that the plasma temperature was intimately associated with the S/N ratio of the chlorine spectral lines.

With respect to theoretical studies, the following studies are significant: Garboczi and Bentz [20] established a cementite microstructure model by simulating the individual minerals of cement particles with the use of pixel dots. Additionally, they investigated the chloride ion diffusion properties of cementite by establishing a transport network, whereby the center of the pixel dots consisted of nodes. However, due to the regular arrangement of the pixel points, the chloride ions could only diffuse in four directions from each node, which is different from their irregular movement in practice. Additionally, because of the large number of pixel points, this method was time consuming and limited by computer technology. Masi et al. [21] developed an ion diffusion model composed of purely physical–chemical equations by exploiting the permeation behavior of chloride ions along with hardened cementite adsorption. In their study, all the parameters were inter-independent and did not have any empirical parameters. However, they created the model on the premise that all the concrete pores were filled with water, and so many factors could possibly have affected the chloride diffusion coefficient; these factors were difficult to fully consider with a purely physical–chemical equation. Nagesh et al. [22] studied the process of chloride infiltration in saturated and unsaturated concrete and proposed a mathematical model. They concluded that the ionic diffusion coefficient and the solution diffusion coefficient made the chloride diffusion process nonlinear. In other work, an effective media approach was used for the assessment of the diffusivity of chlorine [23,24,25]. By modeling cementite as a two-phase composite material, Zheng et al. [23,24] were able to determine the chlorine diffusion coefficient of stiffened cementite according to the effective medium and classical percolation theory. They obtained the parameters through experimental calibration and proposed a simple and effective method of projecting the chlorine diffusivity of the cementite. Zhou et al. [25] proposed an effective medium approach for assessing the mature fly-ash cement paste diffusivity of chlorine; they also used this approach to model a two-phase composite material and analytically deduce the chloride diffusion coefficient.

Brownian motion has been studied by several researchers [26,27,28,29], and from a microscopic point of view, diffusion has been defined as a process caused by ions that are suspended in a solution and performing irregular Brownian motion. The nature of diffusion is a statistical result of the irregular motion of ionic particles. The Einstein–Smoluchowski formulation established the connection between macroscopic ion diffusion behavior and microscopic ion irregular motion. Liu et al. [26] determined the size of representative units of the microstructure of the HYMOSTRUC3D cementite by statistical analysis. They used the random walk method to predict the effective diffusion coefficient of ions for the microstructure model. To avoid walking into a loop or visiting the same location, they specified that ions could walk in only six directions and introduced the self-avoiding walk method, which meant that the same location could not be visited multiple times. When the next step was about to fall in a position that had already been visited, the particle would stagnate at the same place and wait for the next walk, with the walking distance of the step marked as zero. However, the actual process of the random walk cannot be memorized. The self-avoiding walk method can simplify the computation and reduce the computing time, but it can also diminish the accuracy of the simulation, which cannot be called irregular. Zheng et al. [27] and Zhang et al. [28] simulated cement hydration behavior with consideration of the effect of cement particle shape. The cement particles were simplified into round particles and elliptical shapes with different aspect ratios. Brownian motion was implemented in a two-dimensional model to simulate the diffusion behavior of chloride ions in cementite. Both studies were conducted based on a two-dimensional model. The Brownian particles could only move irregularly on the plane, which is significantly different from a true three-dimensional random walk.

The main purpose of this study was to establish a simple, three-dimensional, numerical simulation method based on Brownian motion in order to quantitatively evaluate the chloride ion diffusion coefficient. Compared with two-dimensional simulations or three-dimensional simulations with restricted walking, this method is more realistic and can be used to visualize the cement hydration process and the diffusion behavior of chloride ions in cementite.

## 2. Simulation of the Cement Hydration Process

The cement particles were assumed to be spheres of different sizes; they were located in a cube-shaped simulation cell with a side length of *a*. The cumulative distribution function with respect to the number of cement particles *P_N_* (*d_c_*) [30] was used to calculate the total volume of the simulated cement particles in the cell. Thus, *N_c_* cement particles of different diameters were generated according to a certain *w*/*c*, with the minimum and maximum diameters being $d_{c0}$ and $d_{cm}$, respectively. The generated cement particles were then randomly distributed within the cell, with periodic boundary conditions applied in order to prevent wall effects [31].

(1) $$P_{N}(d_{c})=\frac{\int_{d_{c0}}^{d}x^{\left(\beta -4\right)}\exp(-\alpha x^{\beta })dx}{\int_{d_{c0}}^{d_{cm}}x^{\left(\beta -4\right)}\exp(-\alpha x^{\beta })dx}$$

where $d_{c}$ denotes the diameter; $d_{c0}$ and $d_{cm}$ are the minimum and maximum values of the cement particles, respectively; the coefficient α is 0.038 and is related to the mean diameter; and the dispersion coefficient β is 0.98 for the particle diameter.

(2) $$V_{c}=p_{c}a^{3}$$

(3) $$p_{c}=\frac{1}{1+\frac{\rho_{c}}{\rho_{w}}(w/c)}$$

where $\rho_{w}$ is the water density, and $\rho_{c}$ is the cement density.

**Figure 1:**
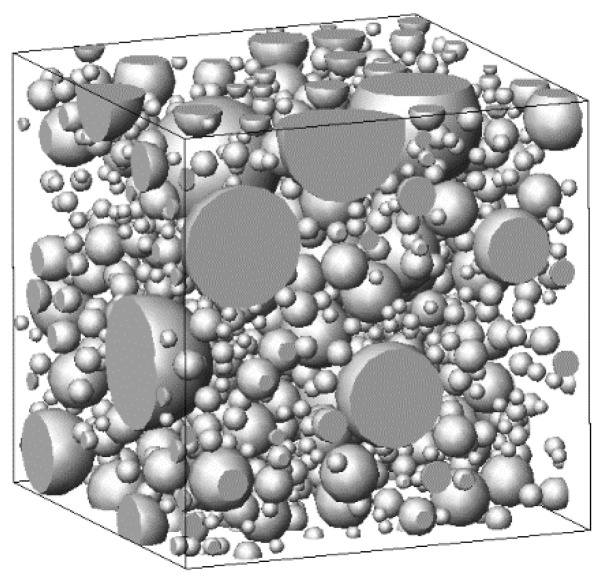
Initial distribution of cement particles.

As an example, with $d_{c0}=1.5\;\mu m$, $d_{cm}=15\;\mu m$, w/c=0.6, and a=50 μm, the original allocation of the simulated cement particles was obtained, as shown in Figure 1.

**Figure 2:**
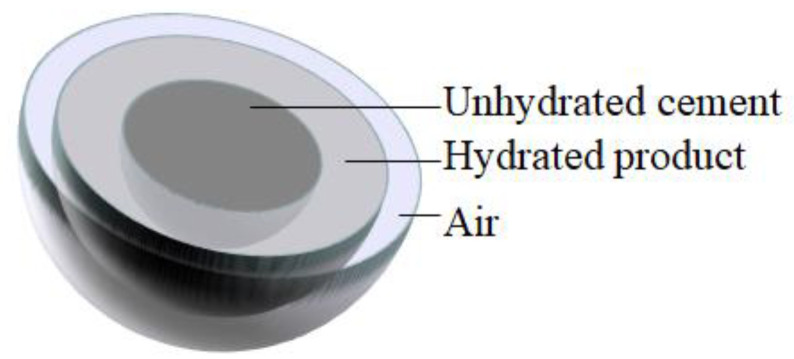
Hydration state of single cement particles.

**Figure 3:**
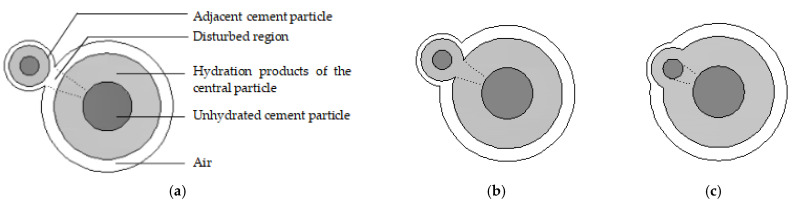
Interference between cement particles: (**a**) from air, (**b**) from hydration product gel, and (**c**) from unhydrated cement particle.

Cement hydration is a complicated procedure that is affected by many factors. The state of a single cement particle at a certain moment during hydration is shown in Figure 2. It consists of air, hydration products, and unhydrated cement, with radii of $r_{a}(t)$, $r_{o}(t)$, and $r_{i}(t)$, respectively. During multiparticle cement hydration, adjacent cement particles interfere with each other, as shown in Figure 3. Based on the hydration theory [32],

(4) $$\Delta {\bar{r}}_{i}=\Delta tK_{0}{\left[\delta /\delta (t)\right]}^{\beta H[\delta (t)-\delta_{tr}]}$$

where $\Delta r_{i}$ is the radius reduction in the unhydrated cement particles, Δt is the time interval, $K_{0}$ is the reaction rate, $\delta (t)=r_{o}(t)-r_{i}(t)$ is the thickness of the gel products at moment *t*, $\delta_{tr}$ is the critical thickness, β is the diffusivity of the gel layer, and H is the step function.

Using $\omega_{0}$, $\omega_{1}$, and $\omega_{2}$ to represent the disturbance degrees of the unhydrated layer, the gel layer, and the air layer of the cement particles, respectively, the reduction values of the volume and radius of the unhydrated cement particles can be determined as follows:

(5) $$\Delta v_{c}=4\pi \omega_{0}[r_{i}^{3}(t)-{\left(r_{i}(t)-\Delta r_{i}\right)}^{3}]/3$$

(6) $$\Delta r_{i}=r_{i}(t)-{\left[(1-\omega_{0})r_{i}^{3}(t)+\omega_{0}{\left(r_{i}(t)-\Delta {\bar{r}}_{i}\right)}^{3}\right]}^{1/3}$$

Furthermore, increases in the radius of the gel layer and air layer are calculated as follows:

(7) $$\Delta r_{o}={\left[\frac{3(k_{0}-1)}{4\pi }\frac{\Delta v_{c}}{\omega_{1}}+r_{o}^{3}(t)\right]}^{1/3}-r_{o}(t)$$

(8) $$\Delta r_{a}={\left[\frac{3k_{1}}{4\pi }\frac{\Delta v_{c}}{\omega_{2}}+r_{a}^{3}(t)\right]}^{1/3}-r_{a}(t)$$

**Figure 4:**
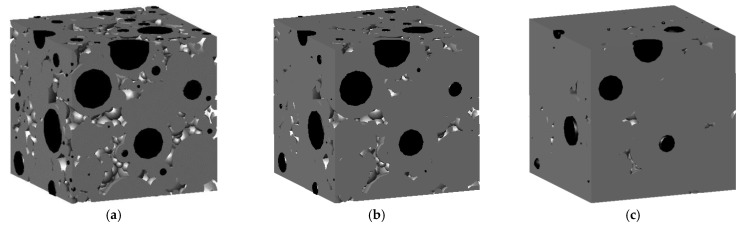
The hydration process: (**a**) 3 d, (**b**) 7 d, and (**c**) 28 d.

The whole hydration process can be simulated with Equations (4)–(8). The unhydrated particles and hydration products of the previously mentioned example, after 3, 7, and 28 d of hydration, are shown in Figure 4. This shows that with the increase in curing age, the hydration continuously proceeded, the radius of the unhydrated cement particles gradually decreased, and the thickness of the gel layer gradually increased. The degree of hydration is the ratio of the hydrated cement particles’ volume to the overall volume at the initial time. Since the ith particle of the unhydrated cement at moment t has a radius of $r_{i,i}(t)$, the degree of hydration can be obtained with the following equation:

(9) $$\alpha_{0}=\frac{V_{c}-\sum_{i=1}^{N_{c}}4\pi r_{i,i}^{3}(t)/3}{V_{c}}$$

**Figure 5:**
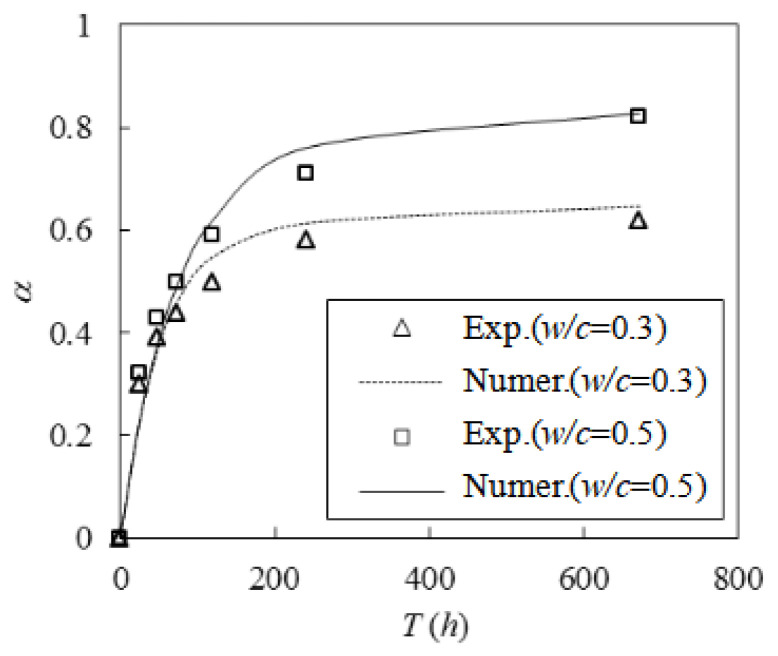
Comparison between the numerical results of hydration and experimental data [33].

To validate the effectiveness of the procedure, we selected a set of experimental data from van Breugel [33] for comparison. The C_3_A, C_4_AF, C_2_S, and C_3_S contents of the cement employed in the experiment were 6.7%, 7.9%, 17.2%, and 56.7%, respectively. The test was conducted at a temperature of 20 °C for 28 d and utilized *w*/*c* values of 0.3 and 0.4. The measured hydration at each moment as well as the simulation results are presented in Figure 5. The results matched well, and when the *w*/*c* was 0.3, the errors between the two results were 7.7% and 0.7% at maintenance ages of 10 d and 28 d, respectively. When the *w*/*c* was 0.4, the errors between the two were 7.7% and 2.3%, respectively. The comparable results indicate that the simulation process was effective.

## 3. Simulation of Chloride Ion Diffusion Behavior

The results of the hydration process revealed that cementite mainly consists of unhydrated cement particles, gel, and pores. The pores are composed of gel pores, which are present in C-S-H, one of the components of gel products, and capillaries, the main pathway for chloride ion diffusion [12]. Therefore, we performed the simulation by simplifying cementite as a composite with a solid phase and a capillary phase.

**Figure 6:**
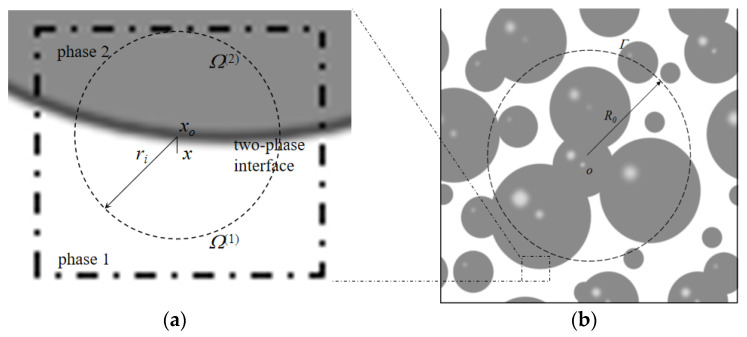
Brownian particles walking in cement paste: (**a**) two-phase interface; (**b**) two-phase cement paste composed of gel and pores.

According to Kim and Torquato [29,34], cementite is a homogeneous matrix that has a chlorine diffusion rate of *D_cp_* when Brownian motion is simulated. Thus, the Brownian particle can be dropped into the simulation cell at an initial position of o, and a sphere with radius *R_0_* can be established, as illustrated in Figure 6b. The average time $\bar{t}(R_{0})$ required for a particle to arrive on the sphere Γ can be obtained using the Poisson equation and by solving for the zero-boundary condition [35].

(10) $$\bar{t}(R_{0})=\frac{R_{0}^{2}}{6D_{cp}}$$

The cement paste can be viewed as an inhomogeneous medium consisting of gel and pores, which have chlorine diffusion rates of zero and *D_p_*, respectively. The Brownian particles can then be dropped into the cell and be permanently trapped if their initial position falls into the gel. Otherwise, a random walk begins when the particle is situated at o in the pore. A maximum sphere with radius r can be constructed with o as the center, just tangential to the nearest cement particle. The Brownian particle then randomly bounces onto the surface. This process is repeated until the particle reaches the threshold Γ for the first time. According to Equation (10), the average arrival time $\bar{t}(r)$ can be determined when the particle is far from the two-phase interface.

(11) $$\bar{t}(r)=\frac{r_{}^{2}}{6D_{p}}$$

The average time can be obtained in a different way when the Brownian particle moves very close to the two-phase intersection; the phases are marked as phase 1 and phase 2, respectively, and are shown in Figure 6a. The Brownian particle is situated at position x of phase 1; $x_{0}$ is the projection point of x from the intersection, and a sphere of radius $r_{j}$ is built with $x_{0}$ as the center. The interface divides the sphere into two sections, $\Omega^{\left(1\right)}$ of volume $V^{\left(1\right)}$ and $\Omega^{\left(2\right)}$ of volume $V^{\left(2\right)}$. Since Brownian particles cannot enter phase 2, the probability of remaining in phase 1 is expressed as $p_{1}=1$. According to Kim and Torquato [34], the average time required for Brownian particles to arrive at $\Omega^{\left(1\right)}$ can be determined with the following equation:

(12) $$\bar{t}(r_{j})=\frac{r_{j}{}^{2}}{6D_{p}}\frac{V^{\left(1\right)}+V^{\left(2\right)}}{V^{\left(1\right)}}\left[1+\frac{1}{2}{\left(\frac{r_{x}}{r_{j}}\right)}^{2}-\frac{1}{2}\sum_{m=0}^{\infty }C_{2m+1}{\left(\frac{r_{x}}{r_{j}}\right)}^{2m+1}\right]$$

where $0\leq r_{x}\leq r_{j}$.

(13) $$C_{2m+1}=\frac{{\left(-1\right)}^{m+1}(2m)!}{2^{2m+1}{\left(m!\right)}^{2}}\frac{3(4m+3)}{\left(2m+1)(m+2)(m+1\right)}$$

Based on Equations (11) and (12), the average time $\bar{t}(R_{0})$ required to arrive at the sphere Γ can be expressed as follows:

(14) $$\bar{t}(R_{0})=\sum_{}\frac{r_{}^{2}}{6D_{p}}+\sum_{}\bar{t}(r_{j})$$

The chlorine diffusion rate of cement paste is defined by a combination of Equations (10) and (14), as expressed by the following equation:

(15) $$D_{cp}=\frac{R_{0}^{2}}{\sum_{}\frac{r_{}^{2}}{D_{p}}+6\sum_{}\bar{t}(r_{j})}$$

In order to be able to use Equation (15) to estimate the chlorine diffusion coefficient of the cement paste, the coefficient of the pore solution is required. Pivonka et al. [12] derived *D_p_*—the diffusion rate of chlorine ions, which is equivalent to 1.07 × 10^−10^ m^2^/s for the pore solution—by comparing the theoretical formula with the experimental results.

**Figure 7:**
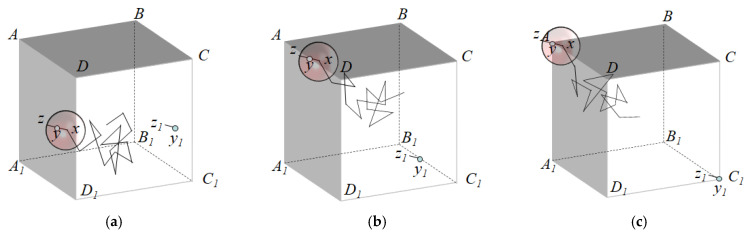
Periodic boundary conditions for Brown particles in the vicinity of the boundary: (**a**) face, (**b**) edge, and (**c**) vertex.

In this simulation, periodic boundary conditions were employed to prevent the Brownian particles from leaving the cell, which would have occurred due to the limitations of the simulation cell. As can be seen in Figure 7, when the Brownian particle arrives at the vicinity of the face *ADD*_1_*A*_1_, the radius *r* required for walking can exceed the cell, in which case the line segment *yz* that exceeds the cell is translated to the line segment *y*_1_*z*_1_ on the right side, at a distance of *a*. When the Brownian particle reaches the edge *AD*, the line segment *yz* that exceeds the cell is translated to the line segment *y*_1_*z*_1_ to its bottom right, at a horizontal and vertical distance *a* from *yz*. Furthermore, as the Brownian particle approaches the vicinity of the vertex *A*, the line segment *yz*, which exceeds the cell, is translated to the line segment *y*_1_*z*_1_, with a horizontal, vertical, and in-depth distance *a* from *yz* at the lower-right vertex. The same process is performed for the other 5 faces, 11 edges, and 7 vertices.

**Figure 8:**
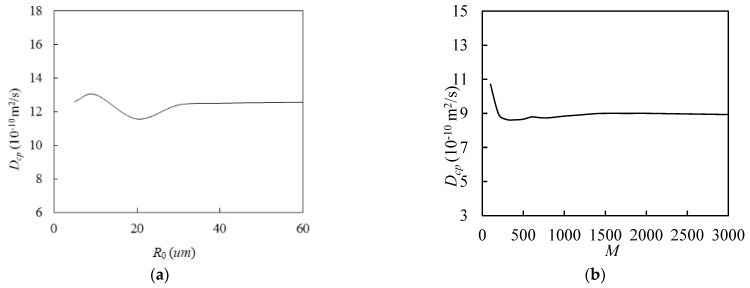
Effect of *R_0_* and *M* on the chloride diffusivity of cement paste: (**a**) effect of *R_0_*; (**b**) effect of *M*.

**Figure 9:**
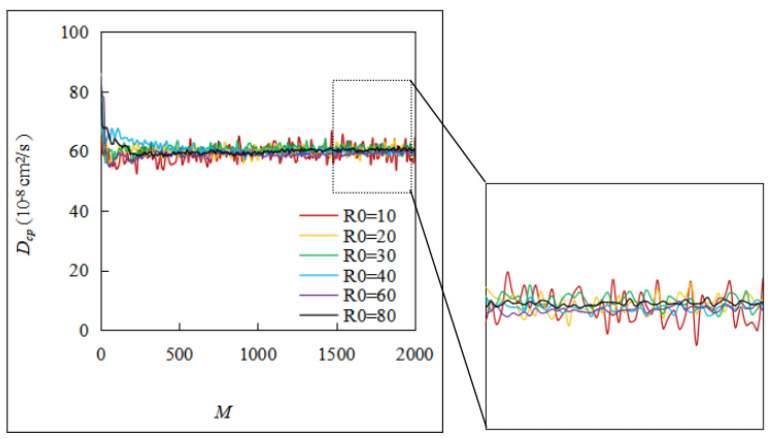
Determination of the values of *M* and *R_0_*.

The larger the predetermined *R_0_*, the longer the particles travel in the simulation cell, and the more accurate the chloride diffusivity of the cement paste is, which obviously requires more calculation time. Additionally, the finite cell size and the initial random seed number can have some effect on the results. The ergodicity assumption can be used to overcome this limitation [36], that is, by taking the average value of the chlorine diffusion rate of the cement slurry *D_cp_*, which is obtained from *M* simulations. To obtain more reasonable *R_0_* and *M*, we attempted to simulate the previously mentioned examples. As shown in Figure 8a, *D_cp_* first fluctuated as *R_0_* increased, and it stabilized as *R_0_* exceeded 40 µm. Similarly, as shown in Figure 8b, *D_cp_* showed some fluctuations when *M* was small, but it converged when *M* exceeded 1500. Therefore, the following simulations used an *R_0_* with a value of 40 and an *M* with a value of 2000. In addition, as can be seen in Figure 9, when *M* was small, *D_cp_* fluctuated sharply. When *R_0_* was small, *D_cp_* tended to gradually smooth out as *M* increased, but this is not obvious. When *R_0_* increased to 30 and above, *D_cp_* gradually converged as *M* continued to increase. The larger the value of *R_0_*, the more obvious the convergence trend. By fixing *M* = 2000 and taking the average value of *D_cp_* when *R_0_* is equal to 80 as the benchmark, the relative errors of the average values of *D_cp_* when *R_0_* is 10 and 40 are calculated to be 6.3% and 0.9%, respectively. Therefore, it is acceptable to take *R_0_* with a value of 40 and *M* with a value of 2000.

## 4. Verification and Discussion

**Figure 10:**
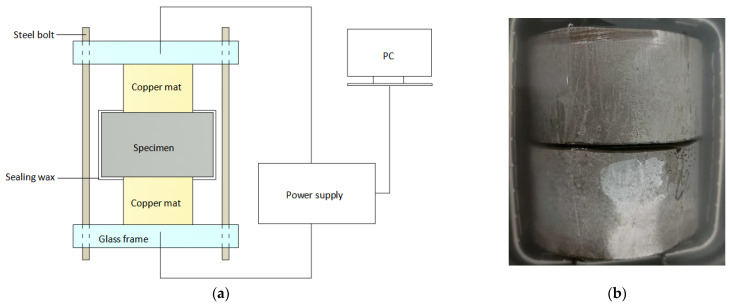
The acceleration equipment and the test specimen: (**a**) equipment; (**b**) test specimen.

**Figure 11:**
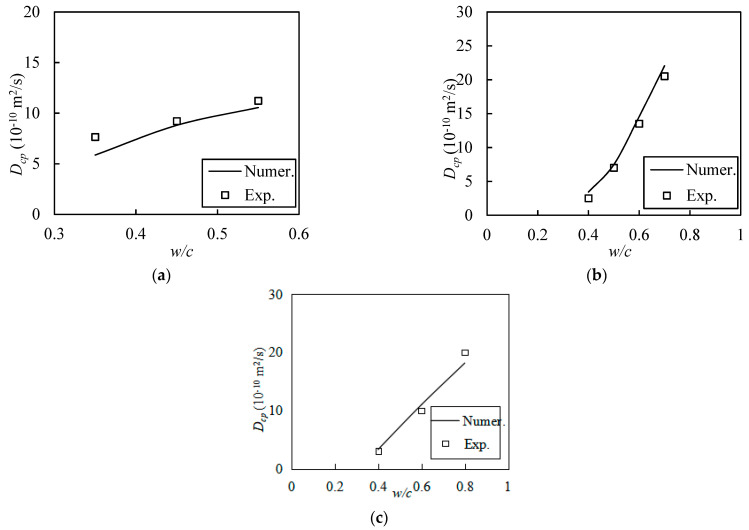
Comparison of numerical results with the experimental results: (**a**) from self-conducted experiments, (**b**) from MacDonald and Northwood [6], and (**c**) from Tang and Nilsson [37].

To verify the numerical simulation results, a set of tests were carried out using the NEL method [8]. The acceleration equipment is shown in Figure 10a. The cement paste specimen was poured into Φ100 × 100 mm cylinders; Portland cement P-O 42.5 (Zhejiang Qianchao Cement Factory, Hangzhou, China) was used, with *w*/*c* values of 0.35, 0.45, and 0.55. After 24 h of standard curing, the specimen was released from the mold and then placed in water at 23 ± 2 °C for 28 d. To avoid the negative influence of factors such as the floating slurry layer, the specimen was transformed after 28 d of curing. The test specimen size was reduced to Φ100 × 50 mm, as shown in Figure 10b, by cutting off 25 mm from both ends. Then, it was subjected to the vacuum saturation salting process. To ensure that the sample would be fully saturated, the time and pressure during the specimen saturation salting process were strictly controlled. The agreement between the experimental and simulation results can be seen in Figure 11a.

The properties of the simulated cell were set so that its side length was *a* = 50 µm; the maximum and minimum diameters of the cement particles were $d_{cm}=15\;\mu m$ and $d_{c0}=1.5\;\mu m$; the *w*/*c* had values of 0.35, 0.45, and 0.55; and the chlorine diffusion rate in the cementite pore solution was $D_{p}=1.07\times 10^{-10}$ m^2^/s. As shown in Figure 11a, the relative differences between the numerical results and the experimental data that correspond to the *w*/*c* values are 23.3%, 4.5%, and 5.8%, respectively. The results confirm the effectiveness of the random walk method.

To further validate the effectiveness of the approach, two sets of experimental data obtained by MacDonald et al. [6] and Tang et al. [37] were selected for comparison. MacDonald et al. [6] used ordinary Portland cement, with *w*/*c* values of 0.4, 0.5, 0.6, and 0.7. The cement paste samples had a cylindrical form, with a diameter of 50 mm and a height of 300 mm; furthermore, the cylinders were demolded after 24 h and then placed in a conservation room, at a temperature of 23 ± 2 °C and a humidity of 100%, for 56 d. The consistency of the two sets of results is illustrated in Figure 11b, whereby the relative errors were 26.9%, 6.4%, 7.3%, and 7.0% for *w*/*c* values of 0.4, 0.5, 0.6, and 0.7, respectively.

In Tang and Nilsson’s experiment [37], which they carried out with *w*/*c* values of 0.4, 0.6, and 0.8 at room temperature, cement paste specimens were soaked in a calcium hydroxide solution for 90 days, which was then accelerated by applying an electric field of 30 V in order to diffuse the chloride ions. In comparison, the temperature used for the simulation in this study was 25 °C, and the other conditions match those of the experiment outlined above. The discrepancies between the two sets of results are presented in Figure 11c. The relative errors between them are 7.0%, 9.1%, and 10.2% for *w*/*c* values of 0.4, 0.6, and 0.8, respectively. The comparison of these two sets of results reaffirms the validity of the method proposed in this paper.

**Figure 12:**
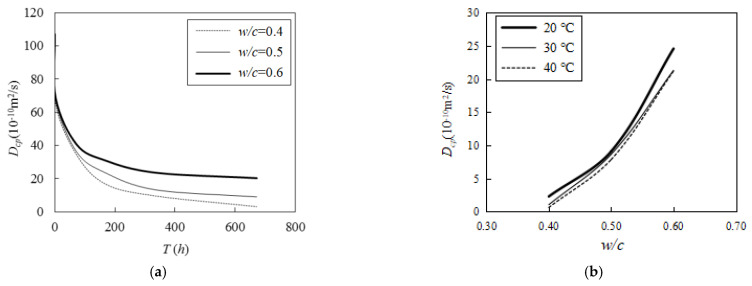
Factors affecting the chloride diffusivity of cement paste: (**a**) effect of curing age; (**b**) effect of curing temperature.

As could be observed in the numerical simulations, with other conditions kept constant, the chlorine diffusion rate of the cement paste remarkably decreased with the augmentation of the curing age (see Figure 12). When the *w*/*c* was set to 0.4, 0.5, and 0.6, the diffusion rate decreased by 95.3%, 86.8%, and 65.7%, respectively, at the age of 28 d as compared with the age of 3 h. By contrast, the chlorine diffusion rate of the cement paste decreased as the curing temperature increased, and it substantially increased as the *w*/*c* increased. This demonstrates that a lower *w*/*c* leads to a lower chloride ion diffusion coefficient.

According to the results of the numerical simulation, as the curing temperature increased from 20 °C to 40 °C, the chlorine diffusivity decreased by 3.59, 1.15, and 1.16 times (see Figure 12b); in addition, the *w*/*c* varied, with values of 0.4, 0.5, and 0.6, respectively. Further, the chlorine diffusivity increased by 3.96 and 7.87 times as the *w*/*c* increased from 0.4 to 0.5 to 0.6 (see Figure 12a). This proves that increasing the maintenance temperature can effectively reduce the diffusion coefficient of chloride ions.

As mentioned before in Figure 4, with the increase in curing age, the hydration continuously proceeded, the radius of the unhydrated cement particles gradually decreased, and the thickness of the gel layer gradually increased. This means the pores became less and less, which leads to a poor interconnectivity. The increase in curing temperature within a certain extent contributes to the better hydration of cement particles. The decrease in *w*/*c* increases the total number of cement particles, resulting in more cement particles participating in hydration. Both effectively reduce the porosity or pore connectivity in cement paste, reduce the migration channels for chloride ions, and lower the diffusion coefficient of chloride ions.

## 5. Conclusions

A simple, three-dimensional, numerical simulation method based on Brownian motion was established to determine the chloride ion diffusion coefficients of cementitious materials. Computer techniques were used to simulate and reconstruct the microstructure of cement paste at a given *w*/*c.* The generation, distribution, and hydration processes of the cement particles were simulated, and the cement paste was eventually reduced to a two-phase material with periodicity. The chloride diffusivity of cement slurry was estimated using a random walk method based on Brownian motion, with periodic boundary conditions applied on the movement. The simulation results indicate that the cement paste chlorine diffusivity gradually converged with the occupation of the total mean-square displacement of the Brownian particles and the number of simulations. Based on the numerical simulations, the impacts of the *w*/*c*, curing temperature, and curing age on the cement paste chlorine diffusivity were discussed. The chloride diffusivity increased with the increase in the *w*/*c*, and it decreased as the curing age and temperature increased. Eventually, the efficiency of the approach was demonstrated by using multiple data sets obtained from the literature or our experiment. This three-dimensional numerical simulation method can replace the experimental method and reduce the time and cost involved in determining the chloride ion diffusion coefficients of cementitious materials. As computer technology advances, the cost and time will be further reduced. In addition, this method can provide ideas for studying the transport properties of other materials or other ions.

## Data Availability

All data, models, or code generated or used during the study are available from the corresponding author upon request.
